# Redox Status and Neuro Inflammation Indexes in Cerebellum and Motor Cortex of Wistar Rats Supplemented with Natural Sources of Omega-3 Fatty Acids and Astaxanthin: Fish Oil, Krill Oil, and Algal Biomass

**DOI:** 10.3390/md13106117

**Published:** 2015-09-28

**Authors:** Tatiana G. Polotow, Sandra C. Poppe, Cristina V. Vardaris, Douglas Ganini, Maísa Guariroba, Rita Mattei, Elaine Hatanaka, Maria F. Martins, Eduardo F. Bondan, Marcelo P. Barros

**Affiliations:** 1 Postgraduate program in Health Sciences, Institute of Physical Activity and Sports Sciences (ICAFE), Cruzeiro do Sul University, R. Galvao Bueno, 868, Building B, 13th floor, Sao Paulo SP 01506000, Brazil; E-Mails: tatianapolotow@gmail.com (T.G.P.); scpoppe@uol.com.br (S.C.P.); crisvardaris@gmail.com (C.V.V.); douganini@uol.com.br (D.G.); maisa_bizie@hotmail.com (M.G.); elaine.hatanaka@cruzeirodosul.edu.br (E.H.); 2 Free Radical Metabolism Group, Laboratory of Toxicology and Pharmacology, National Institute of Environmental Health Sciences, NIEHS, Research Triangle Park, NC 27709, USA; 3 Department of Psychobiology, Federal University of Sao Paulo (UNIFESP), Sao Paulo SP 04023062, Brazil; E-Mail: matteipersoli@gmail.com; 4 Department of Environmental and Experimental Pathology, Paulista University (UNIP), Sao Paulo SP 04026002, Brazil; E-Mails: fa3mterra@terra.com.br (M.F.M.); bondan@uol.com.br (E.F.B.); 5 Program in Veterinary Medicine, Biological Sciences and Health (CBS), Cruzeiro do Sul University, Sao Paulo SP 01506-000, Brazil

**Keywords:** DHA, EPA, carotenoid, antioxidant, hormesis, brain, aging, Alzheimer, Parkinson, senescence

## Abstract

Health authorities worldwide have consistently recommended the regular consumption of marine fishes and seafood to preserve memory, sustain cognitive functions, and prevent neurodegenerative processes in humans. Shrimp, crabs, lobster, and salmon are of particular interest in the human diet due to their substantial provision of omega-3 fatty acids (*n*-3/PUFAs) and the antioxidant carotenoid astaxanthin (ASTA). However, the optimal ratio between these nutraceuticals in natural sources is apparently the key factor for maximum protection against most neuro-motor disorders. Therefore, we aimed here to investigate the effects of a long-term supplementation with (*n*-3)/PUFAs-rich fish oil, ASTA-rich algal biomass, the combination of them, or krill oil (a natural combination of both nutrients) on baseline redox balance and neuro-inflammation indexes in cerebellum and motor cortex of Wistar rats. Significant changes in redox metabolism were only observed upon ASTA supplementation, which reinforce its antioxidant properties with a putative mitochondrial-centered action in rat brain. Krill oil imposed mild astrocyte activation in motor cortex of Wistar rats, although no redox or inflammatory index was concomitantly altered. In summary, there is no experimental evidence that krill oil, fish oil, oralgal biomass (minor variation), drastically change the baseline oxidative conditions or the neuro-inflammatory scenario in neuromotor-associated rat brain regions.

## 1. Introduction

Many motor and cognitive neurodegenerative disorders, such as Alzheimer’s (AD), Parkinson’s (PD), and Huntington’s (HD) diseases, have been consistently associated to oxidative stress in the central nervous system (CNS) and other checkpoints along the neuromotor circuit [1]. The human brain is particularly susceptible to oxidative stress for several reasons, including: (i) massive reactive oxygen/nitrogen species (ROS/RNS) production by intense O_2_ consumption and mitochondrial activity in neurons; (ii) high concentrations of oxidation-sensitive polyunsaturated fatty acids in neuronal membranes; (iii) accumulation of redox-active iron ions in many brain sections; (iv) auto-oxidation of some neurotransmitters to generate neurotoxic compounds, such as 6-hydroxydopamine (from dopamine); and (v) low catalase activity in most brain regions, leading to high H_2_O_2_ susceptibility [2,3]. Despite the incessant cause-or-consequence debate, oxidative stress-linked (neuro) inflammation is also considered a prominent contributor to the pathogenesis of many CNS diseases [4,5,6]. Experimental animal models and patients with AD, PD, and HD show classic features of inflammation in the brain, including phagocyte activation, increased synthesis and release of proinflammatory cytokines, and complement activation [6].

Several neuronal systems are involved in body motion, but the cerebellum is especially linked to the voluntary nervous system. The cerebellum controls the activation, timing, and coordination of distinct muscle groups during body motion. In addition, cerebellar circuits exchange continual neuromotor information to match the intended-executed responses, eliciting fine motor adjustments for proper movements [7]. Interestingly, aging-related motor dysfunctions were associated with an increase in cerebellar markers of lipid and DNA oxidation [8,9]. Excitotoxicity, increased Ca^2+^ dynamics and ROS/RNS formation play important roles in cerebellar toxicity based on several studies using oxyradical promoters, such as 2-chloropropionic acid, methyl mercury, *etc.* [10,11]. Although other focal sensorimotor areas of the human brain are truthfully important for motor coordination, such as the supplementary motor area, and pre- and postcentral gyri [12], the link between cerebellar activity and oxidative stress might be also determinant to assure a better life quality of the elderly population, exercising people or even athletes during their professional careers [13,14].

The consumption of marine fish and seafood has been associated with mental health, the prevention of neurodegenerative processes, and the maintenance of cognitive functions during human lifespan. Most of the neurological benefits provided by regular seafood consumption come from an adequate uptake of omega-3 polyunsaturated fatty acids, (*n*-3)/PUFAs, and the antioxidant carotenoid astaxanthin (ASTA) [15]. In the nervous system, cell membranes contain relatively high concentrations of (*n*-3)/PUFAs, such as docosahexaenoic acid (DHA) and eicosapentenoic acid (EPA) [16]. Studies from our group demonstrated that the combination of ASTA and (*n*-3)/PUFAs (in fish oil; FO) surpasses the cognitive benefits observed in FO-fed Wistar rats due to an additional antioxidant protection provided by ASTA at the cathecolaminergic-rich anterior forebrain of rats (associated with the anxiety behavior) [17]. In fact, higher levels of lipid and protein oxidation were observed in the anterior forebrain of animals when treated only with FO, although positive anxiolytic effects were concomitantly observed. Controversial studies have given rise to the hypothesis that unbalanced (*n*-3)/PUFAs consumption could drastically affect fluidity, permeability, and hydrophobicity of the neuronal membrane leading to higher oxidation sensitivity, impaired signal transduction and lower neurotransmission efficiency [18,19]. On the other hand, ASTA possesses powerful antioxidant and anti-inflammatory activities already evidenced *in vitro*, in animal models, and in humans [20,21,22,23]. In humans, ASTA has been suggested to perform several beneficial functions including protection against UV-light photo-oxidation in skin cells, control of carcinogenic processes, prophylaxis/regression of stomach ulcers caused by *Helicobacter pylori* infection, delaying exercise fatigue, and promotion of liver, heart, eye, joint, and prostate health [23,24]. Therefore, the beneficial properties of a regular ingestion of (*n*-3)/PUFAs and ASTA apparently arise from the proper balance between these components in foodstuff (or supplements) and their bioavailability.

Among new findings on (*n*-3)/PUFAs nutrition, krill oil has gaining the attention of the medical community [25]. Krill oil (KO) is extracted from the Antarctic microcrustacean *Euphausia superba* and is a rich source of (*n*-3)/PUFAs, including EPA and DHA, phospholipids, and ASTA [26]. Pre-clinical studies have shown that absorption of phospholipid-attached PUFAs into the heart, brain and liver of animals is better than as triacylglycerol molecules (TAG) [27]. Taking that into account, bioavailability favors KO as a better (*n*-3)/PUFA source than FO, since a significant 40% of the total fatty acids attached to phospholipids in KO are EPA and DHA, whereas FO contains mostly TAG [28]. Accordingly, studies with animal models and humans suggest that KO is more effective than FO in reducing cardiovascular risk indexes [29,30]. Moreover, metabolic effects of KO were reported to be similar to those of FO but at lower doses of EPA and DHA in healthy volunteers [31]. Despite the many published studies comparing different (*n*-3)/PUFAs sources, no scientific paper, to the best of our knowledge, has ever compared the effects of FO, ASTA, the combination of them, and KO in terms of redox balances and inflammation in brain regions associated with motor control, as cerebellum, the motor cortex, and corpus callosum. Taking that into account, the aim of this work is to investigate if some natural sources of (*n*-3)/PUFAs and ASTA alter the baseline redox status and neuro-inflammation indexes in animal cerebellum and motor cortex.

## 2. Results

### 2.1. Cerebellum

**Figure 1:**
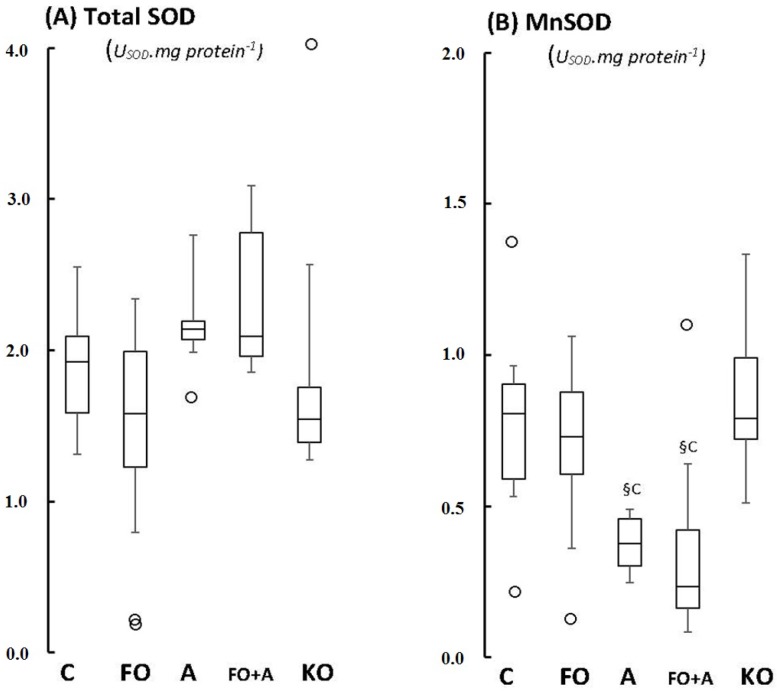
Boxplots of the (**A**) total SOD and (**B**) mitochondrial MnSOD activity in cerebellum of Wistar rats supplemented for 45 days with fish oil (FO, for 10 mg EPA/kg BW + 7 mg DHA/kg BW), algal biomass (A, for 1 mg astaxanthin (ASTA)/kg BW), fish oil + algal biomass (FO + A, for 10 mg EPA/kg BW + 7 mg DHA/kg BW + 1 mg ASTA/kg BW), and krill oil (KO, for 10 mg EPA/kg BW + 4.7 mg DHA/kg BW + 7.2 μg ASTA/kg BW). (*n* ≥ 6, §c < 0.05). (_°_) Outliner data of the IQR = 1.15 × (Q3 − Q1), where: IQR = interquartile range; Q3 = 75% quartile; and Q1 = 25% quartile.

Figure 1 depicts the box plots of total and manganese-dependent (mitochondrial) SOD activities in the cerebellum of control, FO-, ASTA-, FO + ASTA-, and KO-fed animals (Figure 1A,B respectively). No significant difference in total SOD activity was observed between groups. On the other hand, ASTA supplementation seems to significantly decrease the enzyme activity of MnSOD in cerebellum of rats, since both ASTA- and FO + ASTA-groups showed, respectively, 50% (*p* = 0.0456) and 59.6% (*p* = 0.0279) lower MnSOD activities than control (Figure 1B).

**Figure 2:**
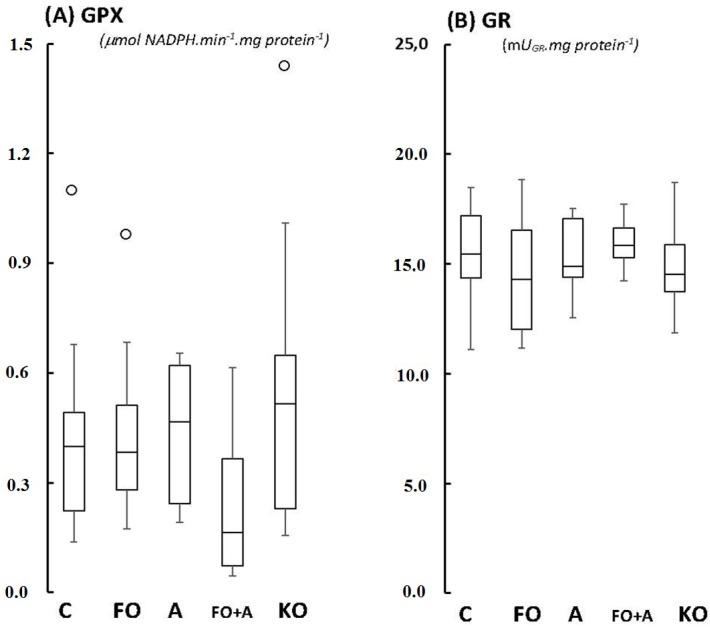
Boxplots of (**A**) glutathione peroxidase, GPX; and (**B**) glutathione reductase, GR, activity in cerebellum of Wistar rats supplemented for 45 days with fish oil (FO, for 10 mg EPA/kg BW + 7 mg DHA/kg BW), algal biomass (A, for 1 mg ASTA/kg BW), fish oil + algal biomass (FO + A, for 10 mg EPA/kg BW + 7 mg DHA/kg BW + 1 mg ASTA/kg BW), and krill oil (KO, for 10 mg EPA/kg BW + 4.7 mg DHA/kg BW + 7.2 μg ASTA/kg BW). (*n* ≥ 6). (_°_) Outliner data of the IQR = 1.15 × (Q3 − Q1), where: IQR = interquartile range; Q3 = 75% quartile; and Q1 = 25% quartile.

**Figure 3:**
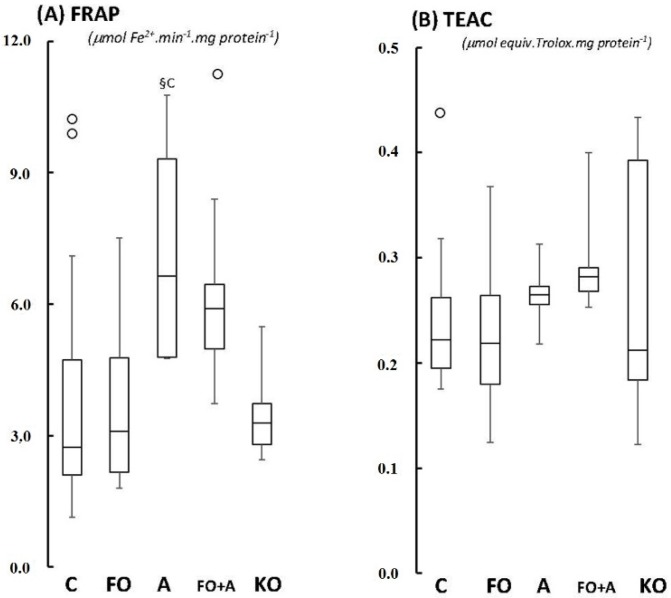
Boxplots of the (**A**) ferric-reducing, FRAP; and (**B**) Trolox-equivalent antioxidant, TEAC, activity in cerebellum of Wistar rats supplemented for 45 days with fish oil (FO, for 10 mg EPA/kg BW + 7 mg DHA/kg BW), algal biomass (A, for 1 mg ASTA/kg BW), fish oil + algal biomass (FO + A, for 10 mg EPA/kg BW + 7 mg DHA/kg BW + 1 mg ASTA/kg BW), and krill oil (KO, for 10 mg EPA/kg BW + 4.7 mg DHA/kg BW + 7.2 μg ASTA/kg BW). (*n* ≥ 6; §c < 0.05). (_°_) Outliner data of the IQR = 1.15 × (Q3 − Q1), where: IQR = interquartile range; Q3 = 75% quartile; and Q1 = 25% quartile.

**Figure 4:**
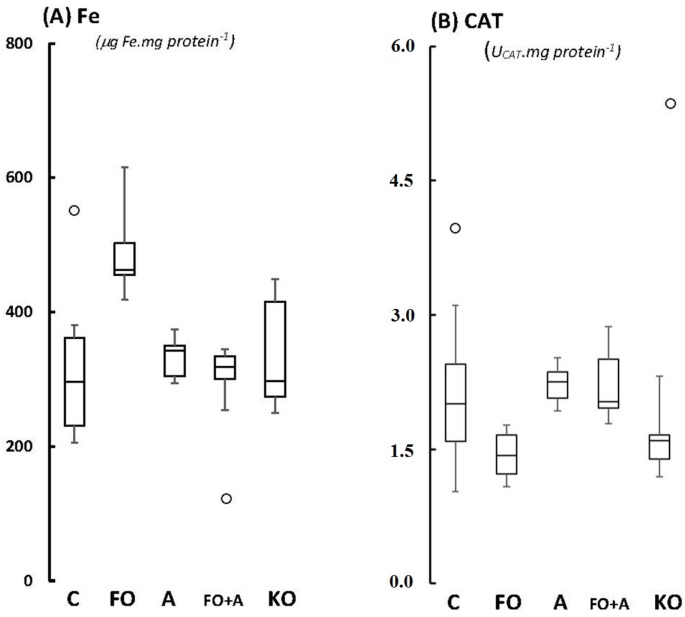
Boxplots of (**A**) iron content; and (**B**) catalase activity, CAT, in cerebellum of Wistar rats supplemented for 45 days with fish oil (FO, for 10 mg EPA/kg BW + 7 mg DHA/kg BW), algal biomass (A, for 1 mg ASTA/kg BW), fish oil + algal biomass (FO+A, for 10 mg EPA/kg BW + 7 mg DHA/kg BW + 1 mg ASTA/kg BW), and krill oil (KO, for 10 mg EPA/kg BW + 4.7 mg DHA/kg BW + 7.2 μg ASTA/kg BW). (*n* ≥ 6). (_°_) Outliner data of the IQR = 1.15 × (Q3 − Q1), where: IQR = interquartile range; Q3 = 75% quartile; and Q1 = 25% quartile.

Glutathione peroxidase (GPX; Figure 2A), glutathione reductase (GR; Figure 2B), Trolox-equivalent Antioxidant Capacity (TEAC; Figure 3B), and catalase activities (CAT; Figure 4A) in cerebellum were unaltered between groups, with *p*-values varying from 0.208 (CAT activities in FO *versus* KO) up to 0.687 (TEAC activities in FO *versus* control). Regarding ferric-reducing activity (FRAP), significant differences were only observed between ASTA-fed and control groups: FRAP was 2.1-fold higher in ASTA-fed group compared to control (Figure 3A). No significant differences were observed in total iron content in cerebellum of rats from any treated group (Figure 4A).

**Figure 5:**
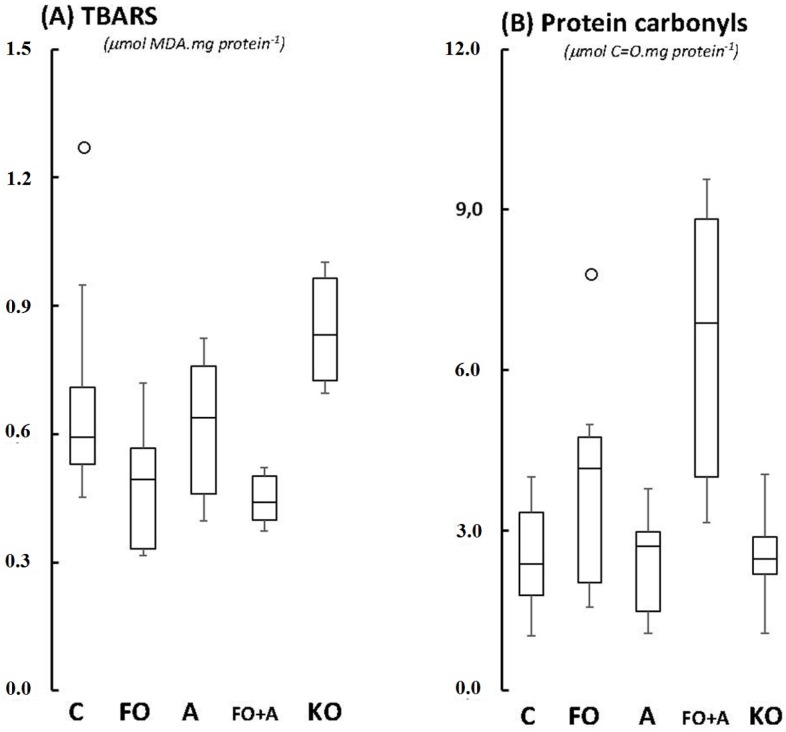
Boxplots of (**A**) thiobarbituric acid reactive substances content, TBARS; and (**B**) protein carbonyls in cerebellum of Wistar rats supplemented for 45 days with fish oil (FO, for 10 mg EPA/kg BW + 7 mg DHA/kg BW), algal biomass (A, for 1 mg ASTA/kg BW), fish oil + algal biomass (FO + A, for 10 mg EPA/kg BW + 7 mg DHA/kg BW + 1 mg ASTA/kg BW), and krill oil (KO, for 10 mg EPA/kg BW + 4.7 mg DHA/kg BW + 7.2 μg ASTA/kg BW). (*n* ≥ 6). (_°_) Outliner data of the IQR = 1.15 × (Q3 − Q1), where: IQR = interquartile range; Q3 = 75% quartile; and Q1 = 25% quartile.

Finally, no significant differences were detected between groups regarding the levels of lipid (TBARS, Figure 5A) and protein oxidation (protein carbonyls, Figure 5B), demonstrating that the observed slight redox baseline changes were not sufficient to induce oxidative modifications in essential biomolecules for cellular survival.

### 2.2. Motor Cortex (Molecular and Granular Layers) and Corpus Callosum

Due to the increasing interest on KO as a potential natural source of both (*n*-3)/PUFAs and ASTA, we decided to extend our study into the effects of KO supplementation in the motor cortex (molecular and granular layers) and corpus callosum of Wistar rats. Therefore, inflammatory cytokines and glutathione-based antioxidant defenses were evaluated in rat brain regions related with neuromotor control. As shown in Table 1, none of the evaluated parameters had baseline levels altered by the 45-day supplementation with KO, which was adjusted for 10 mg EPA/kg BW, 4.7 mg DHA/kg BW, and 7.2 μg ASTA/kg BW.

**Table 1:** Glutathione-based redox parameters and inflammatory cytokines in the motor cortex (molecular and granular layers) and in the corpus callosum regions of Wistar rats supplemented for 45 days with krill oil (equivalent to 10 mg EPA/kg BW, 4.7 mg DHA/kg BW, and 7.2 μg ASTA/kg BW (*n* = 3).

Parameters ^a^	Control	KO	*p* (*t*-Student’s)
GSH (µmoL/mg protein)	71.1 ± 28.6	48.4 ± 9.9	0.085
GSSG (µmoL/mg protein)	39.2 ± 3.9	53.5 ± 26.0	0.180
Reducing power (A.U.)	0.464 ± 0.070	0.334 ± 0.122	0.317
VEGF (pg/mg protein)	39.3 ± 8.3	37.5 ± 12.5	0.284
l-SEL (pg/mg protein)	67.9 ± 65.0	52.2 ± 24.7	0.295
CINC1 (ng/mg protein)	3.76 ± 0.84	5.40 ± 3.49	0.060
MIP1a (ng/mg protein)	15.2 ± 5.3	12.8 ± 3.5	0.491
TNF-α (ng/mg protein)	17.5 ± 8.4	15.0 ± 2.2	0.371
IL-6 (ng/mg protein)	1.71 ± 0.91	1.32 ± 0.41	0.178
IL-1β (ng/mg protein)	1.30 ± 0.81	1.18 ± 0.47	0.388

^a^ Reduced glutathione, GSH; oxidized glutathione, GSSG; vascular endothelial growth factor, VEGF; l-selectin, l-SEL; cytokine-induced neutrophil chemoattractant-1, CINC-1; macrophage inflammation protein, MIP1α; tumor necrosis factor-alpha, TNFα; interleukin-6, IL-6; and interleukin-1-beta, IL-1β; astaxanthin, ASTA.

**Figure 6:**
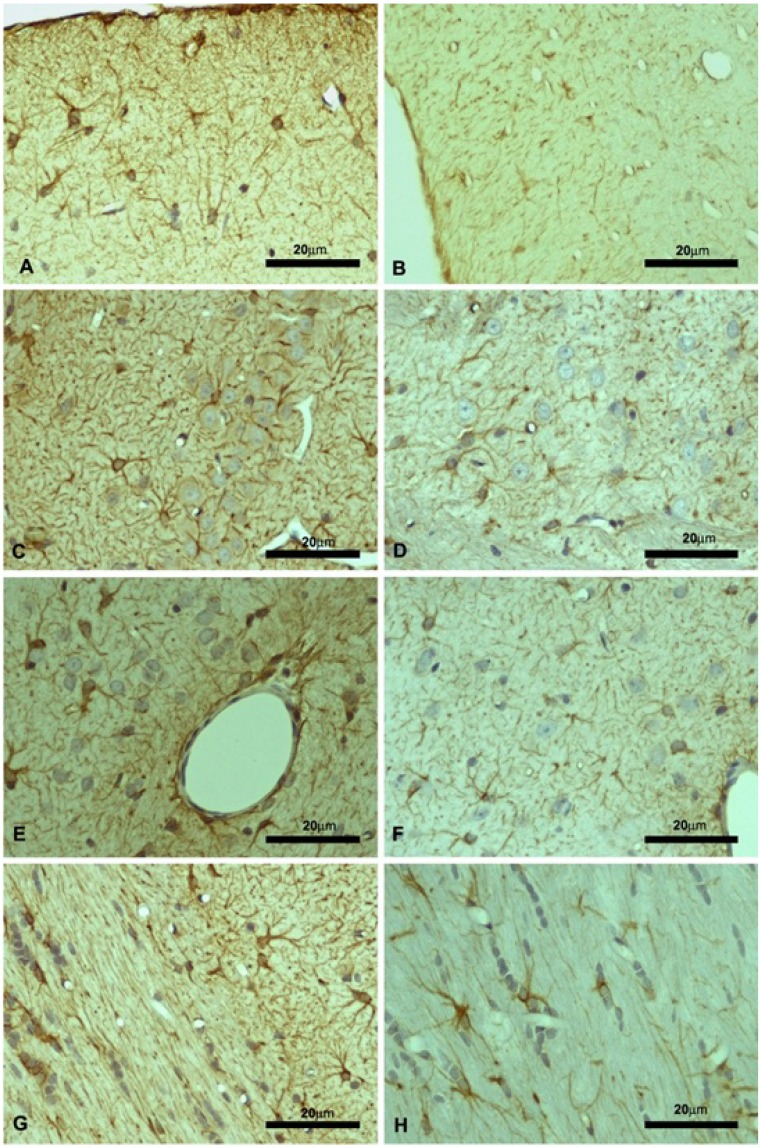
Glial fibrillary acidic protein (GFAP) expression in brain regions of Wistar rats that received krill oil (KO, for 10 mg EPA/kg BW + 4.7 mg DHA/kg BW + 7.2 μg ASTA/kg BW) is expressed in panels (**A**,**C**,**E**,**G**), and control in panels (**B**,**D**,**F**,**H**). Specific regions: Molecular layer of the motor cortex (**A**,**B**); granular layer of the motor cortex (**C**,**D**); perivascular area near the granular layer (**E**,**F**); and corpus callosum (**G**,**H**) (*n* = 3).

Moreover, glial fibrillary acidic protein (GFAP)-positive cells were identified via light microscopy by the intense brown staining in their cytoplasm and were considered astrocytes (Figure 6). Quantified areas (in µm^2^) of GFAP-staining for the animals that received or not KO are shown in Table 2. Significant difference (*p* < 0.05) was observed in GFAP expression by astrocytes between both KO and control groups in all considered areas.

**Table 2:** Quantified staining areas, in µm^2^, for glial fibrillary acidic protein (GFAP) expression in the motor cortex (molecular and granular layers) and in the corpus callosum regions of Wistar rats supplemented for 45 days with krill oil, equivalent to 10 mg EPA/kg BW, 4.7 mg DHA/kg BW, and 7.2 μg ASTA/kg BW. Total scanned area of 302,952.5 µm^2^ (*n* = 3).

	Control			KO		
Area ^a^	Mol. Layer	Gran. Layer	C. callosum	Mol. Layer	Gran. Layer	C. callosum
1	5481.6	4391.7	4928.8	8435.5	6582.6	8374.7
2	4932.7	3837.9	3978.5	7345.4	7543.1	7945.5
3	4637.7	3291.8	4185.6	7581.5	6908.4	7531.9
4	4147.2	4317.8	3281.7	8190.2	6683.8	7435.8
5	3184.6	3928.8	4921.8	8284.7	7385.6	8528.6
6	4841.1	4193.8	3826.9	7858.5	7483.5	6836.7
7	3802.6	4928.8	4167.8	7593.0	6538.5	6638.5
8	4475.9	5102.3	3475.9	7395.7	7438.5	7578.9
9	3179.5	4294.9	4201.1	8193.6	8583.6	7538.6
10	3548.2	4291.7	3739.7	8530.5	8147.7	6625.8
Mean (±SD)	4223.1 (±782.5)	4258.0 (±517.4)	4070.8 (±543.0)	7940.9 ^§^ (±440.4)	7329.5 ^§^ (±675.3)	7503.5 ^§^ (±665.1)

^a^ Molecular layer of motor cortex, Mol. Layer; granular layer of motor cortex, Gran. Layer; corpus callosum, C. callosum. ^§^ Significant difference compared to the same region in control group (*p* < 0.05).

## 3. Discussion

Although many redox and inflammatory parameters were proposed here, we, in fact, did not expect to observe substantial changes of their values in cerebellum, motor cortex or corpus callosum of Wistar rats fed with natural sources of (*n*-3)/PUFAs and/or astaxanthin, since no oxidative challenge was actually imposed to experimental animals. We simply aimed here to investigate any baseline redox/inflammatory peculiarity concerning the long-term supplementation with (*n*-3)/PUFAs and/or astaxanthin in motor control brain regions. Definitely, more answers regarding the efficiency of antioxidant therapies provided by marine nutraceuticals should come out by reproducing the experiments here in well-established neurodegenerative animal models, such as Alzheimer’s and Parkinson’s diseases [32,33] or age-related sarcopenia models [34]. All these neurodegenerative processes are marked by alterations in the free radical metabolism and accumulation of oxidized products and/or oxylipid adducts during disease progression in humans and animals [35].

Significant redox changes were only observed in cerebellum of Wistar rats after 45 days of ASTA supplementation: decreases of mitochondrial MnSOD activity in ASTA (−50%) and FO + ASTA-fed animals (−60%), and a 2-fold increase of antioxidant capacity (FRAP test) in ASTA-fed rats (Figure 1B and Figure 2A, respectively). Evidence shows that ASTA properly crosses the blood-brain barrier to reach and distribute (homogenously or heterogeneously?) within different mammalian brain regions [36,37]. Moreover, many authors have already proposed a mitochondrial-targeted action of ASTA in most mammalian models: (i) ASTA reverted the pro-apoptotic effects of MPP^+^ in culture cells via upregulation of Bcl-2 and downregulation of Bax and α-synuclein protein expression, and concomitant inhibition of caspase-3 activation [38]; (ii) ASTA promoted mitochondrial β-oxidation by favoring the colocalization of fatty acid translocase (FAT/CD36) with carnitine-palmitoyltransferase I (CPT I) during aerobic-type exercises [39]; (iii) ASTA significantly inhibited apoptosis, mitochondrial dysfunction and intracellular ROS/RNS over production induced by 6-hydroxydopamine treatment in cultured SH-SY5Y cells [40]; and (iv) ASTA treatment optimized mitochondrial function in leukocytes of both young and aged dogs by adjusting ATP synthesis with cytochrome c oxidoreductase activity [41]. The downregulation of mitochondrial MnSOD activity in cerebellum of ASTA- and FO + ASTA-fed animals (Figure 1B) could be partially explained by such an *in situ*, organelle-specific, antioxidant protection provided by ASTA. Although the TEAC index was not significantly altered, FRAP activities in cerebellum of ASTA- and FO + ASTA-groups corroborate this hypothesis. In addition, it is tempting to suggest that ASTA supplementation could positively influence neuron function by controlling mitochondrial activity and, concomitantly, regulating ROS/RNS production, intracellular redox status and possibly Ca^2+^ dynamics necessary for synaptic communication. Further studies are obviously necessary to confirm this hypothesis.

Based on our calculations and analytical determinations, KO supplementation provided 10 mg EPA/kg BW, 4.7 mg DHA/kg BW, and 7.2 μg ASTA/kg BW, daily, to experimental animals. The ASTA content present in KO supplement is almost 140-fold lower than that offered in FO + A group, although DHA content in KO was also slightly lower than in FO + A group (7 mg DHA/kg BW in FO + A group; Experimental Section). Therefore, it is not surprising that both FO and KO groups here showed mostly identical redox indexes in cerebellar samples after 45 days of supplementation. In fact, metabolic effects of KO were reported to be similar to those of FO but at lower doses of EPA and DHA in healthy volunteers [31]. Unfortunately, we did not measure plasmatic concentration of ASTA during the supplementation period, which could clarify if the minor content of ASTA in KO supplement (7.2 μg/kg BW) was actually absorbed by animals in any extent. Few significant changes (and other mere statistical tendencies) cannot confirm abrupt different redox scenarios upon FO + A supplementation compared to ASTA-fed group in cerebellar samples here.

Previous studies from our group demonstrated that identical doses of ASTA and FO as applied here (also for 45 days) actually resulted in some similar redox changes at the cathecolaminergic-rich anterior forebrain of Wistar rats, a brain region associated with anxiety behavior [17]. Concerning absolute values: (i) FO and FO + A groups showed approximately 60% lower mitochondrial MnSOD activity than control in the anterior forebrain of Wistar rats (previous publication), whereas FO + A and A groups here (but not FO group) displayed exactly the same reduction in cerebellum; (ii) as shown in anterior forebrain from last publication, GPX or GR activities were absolutely unaltered here, considering any applied supplementation; (iii) baseline antioxidant capacities (TEAC and FRAP) and antioxidant enzyme activities (total SOD, CAT, GPX, and GR) were exactly at the same range in both papers, comparing the different supplementation groups; (iv) increments in iron content were observed in the anterior forebrain of Wistar rats only after FO and FO + A supplementation (approximately 45%), whereas no changes were observed here in cerebellum, but merely statistical tendencies after FO administration (Figure 4A; *p* = 0.0614) [17].

Although very similar oxidative environments were presented in the cerebellum and in the anterior forebrain of Wistar rats (as we published before), we presume that different cathecolamine content between these two brain segments, together with the attested higher iron availability in the anterior cortex, would culminate in the higher susceptibility of the anterior cortex to oxidation, compared to cerebellum, as investigated here [42]. Accordingly, other groups showed that specific brain regions might evoke differential antioxidant responses against oxidative conditions imposed by free radical-promoting agents, such as rotenone and lipopolysaccharide (LPS) [43]. However, the authors perceived lipid peroxidation as a less variable parameter than, for example, GSH levels in such specific brain sections. The difference between patterns of lipid peroxidation and antioxidants was attributed to distinguished content of (*n*-3)/PUFAs in the brain. Undoubtedly, FO (alone or in the presence of ASTA) or KO supplementation, as we performed here, could also affect the ratio between EPA and DHA in different brain regions, as well. Such variability in brain regions to respond against oxidative stress might be pivotal to explain the occurrence of specific neurodegeneration disorders in animal models and humans [43]. Furthermore, frontal cortex, hippocampus, substantia nigra, and hypothalamus of rat brain showed significant neuronal abnormalities (cell death and dysfunction) after an intracranial administration of the free radical-generating agent rotenone, but not the cerebellar nucleus [44]. These data corroborate the hypothesis that cerebellar segment is putatively more resistant to oxidative imbalances than other dopaminergic-rich brain sections [45,46].

A bulk of data from the last few years depicts astrocytes as the major focus of researchers to decipher the neurodegenerative mechanisms at early phases of diseases (pathologies). Activated astrocytes are, thereby, considered as a hallmark of neurodegenerative diseases since these cells release a wide array of pro-and anti-inflammatory cytokines, free radicals, antioxidants, and neurotrophic factors during pathological conditions [47]. Among several signal factors released by activated astrocytes, the expression of glial fibrillary acidic proteins (GFAP) is regarded as one of the most accurate parameters to monitor time-dependence, intensity, and, as properly applied here, altered redox conditions in brain sections [48]. Interestingly, the increased expression of GFAP here suggest that astrocytes were significantly activated in the motor cortex and corpus callosum of Wistar rats treated with KO for 45 days (Figure 6), despite no clear variation being observed in cytokines or antioxidant levels in the same brain regions (Table 1). Mild oxidative conditions that were not properly quantified by thiol-dependent indexes (reduced/oxidized GSH ratio; Table 1) might have provoked activation of astrocytes in the motor cortex of KO-fed animals, although a clear scenario of neuro-inflammation was not evidenced either. Once again, it is worth mentioning that no robust oxidative challenge was actually imposed to rat brains and, therefore, slight variations on redox balances would be only clearly detected depending on the sensibility of the analytical method applied. We presume the GSH/GSSG ratio, in this case, was not sensitive enough for a proper characterization of the mild oxidative condition imposed by long-term supplementation with KO in the motor cortex.

An increasingly relevant “neuro-hormesis” concept states that many health-promoting nutraceuticals actually exert mild pro-oxidant conditions in specific tissues or cells, which is then positively counteracted by induced antioxidant responses [49,50]. Then, the induced antioxidant increment—normally obtained through the Nrf2-Keap2-AREs pathway—would rebalance redox statuses in all exposed tissues and, as a multi component effect in the whole organism, it would chronically bring a general healthy condition for humans taking that nutraceutical [51]. Intracellular redox statuses have been claimed as limiting indexes to determine cellular fates: from a reductive environment through normal, mild or harmful oxidative conditions, a proper cell (if provided with sufficient cellular apparatuses for that) would enter into proliferation/differentiation, sustain its steady-state condition (G0), trigger apoptotic mechanisms or, finally, succumb to necrosis processes [52]. The redox boundaries that limit each cellular stage obviously depend on cell type. Less “flexible” cells, like neurons and glial cells probably have a narrower redox range determining the steady-state condition. Neurons notoriously have lower proliferation capacity, limited conditions to enter through apoptosis, and are also highly susceptible to oxidation, compared to hepatocytes or myocytes, for example. Therefore, even slight redox changes could drastically alter neuron functions and determine morphological and/or cellular modifications. Current knowledge also suggests that synapse turnover (renowned as synaptic plasticity) is modulated, at least in part, by the redox balance at the synaptic site, involving both ROS/RNS and neuroprotective antioxidants [53,54]. Neuroscientists are now shedding light on initial molecular events, especially those related to neuronal reticulum and mitochondrial Ca^2+^-biochemistry, neuronal redox balances, and synaptic plasticity at the early stages of neurodegenerative diseases [55].

## 4. Experimental Section

### 4.1. Chemicals

All purified chemicals were purchased from Sigma-Aldrich Chemical Company (St. Louis, MO, USA), except common laboratory solutions and buffers, which were obtained from Labsynth (Diadema, Brazil).

### 4.2. Natural Sources of (n-3)/PUFAs and Astaxanthin

Fish oil capsules were purchased as Corabion HC 550 supplements (Merck, Relthy Laboratórios Ltda, Indaiatuba, Brazil). We used the nutritional information provided by the manufacturers to plan animal supplementation. All details about the nutrient composition in the raw material are presented in a separated section (Supplementary Materials).

Natural ASTA supplements (AstaREAL A1010) were obtained as a donation from the Swedish company BioReal AB (Gustavsberg, Sweden), a subsidiary of BioReal Inc. (Hawaii, HI, USA) and part of the pharmaceutical Group Fuji Chemical Industry Co. (Toyama, Japan). We extracted ASTA from AstaREAL biomass in acetone in order to quantify the carotenoid content in the provided material (absorbance at 473 nm *versus* a standard curve). We determined ASTA content as (4.1 ± 1.7) mg ASTA/100 mg AstaREAL biomass, similar to the results reported by BioREAL AB [56,57]. An ASTA stock solution of 20 mg AstaREAL/mL (0.82 mg ASTA/mL, based on our calculations) was prepared in 10% Tween-80 aqueous solution (*v*/*v*) by previously grinding AstaREAL biomass in a mortar and dissolving it by mixing manually, with the help of an ultrasound bath, when necessary.

Krill oil was purchased from Mega Red^®^ Extra Strength 500 (Schiff Vitamins, Parsippany, NJ, USA). No information was provided by manufacturers about the ASTA content in Mega Red softgels. Repeating the same procedures used here before (quantifying ASTA in AstaREAL biomass), we measured 0.092 mg ASTA/g krill oil, which was applied in our supplement calculations [58,59].

### 4.3. Animals and Supplementation Protocols

Adult Wistar male rats (*n* = 36), weighing approximately 150 g at the beginning of the study were provided by the Department of Psychobiology, Federal University of São Paulo (UNIFESP), Sao Paulo, Brazil. All animal were housed in Plexiglas cage (4 rats/cage) under standard laboratory conditions: 12 h light/dark cycle; lights on at 7:00 a.m.; (22 ± 1) °C; and *ad libitum* access to water and Purina rat chow. The Ethics Committee for experimental animals from Cruzeiro do Sul University approved all the experimental protocols described here (CE/UCS-194/2011). All experimental procedures were in accordance with the Guide for the Care and Use of Laboratory Animals (1996, published by National Academy Press, 2101 Constitution Ave. NW, Washington, DC 20055, USA). The animals were treated with ASTA, FO, and KO by gavage, 5 days a week, for 45 days. Since different (*n*-3)/PUFA and ASTA compositions are present in FO, ASTAReal, and KO, we decided to normalize the supplementations based on the EPA content: 10 mg EPA/kg BW in animals, following the worldwide recommendations for EPA + DHA daily uptake of 1000 mg EPA + DHA/day (accounting for 14.3 mg EPA + DHA/kg/day in an adult male with 70 kg). Based on that, our FO and FO + ASTA protocols provide 17 mg EPA + DHA/kg/day in experimental animals, and KO supplementation furnishes exactly 14.3 EPA + DHA/kg/day. Single ASTA supplementation was fixed for 1 mg ASTA/kg BW, based on our previous positive results. Natural FO, ASTAReal, and KO products were diluted in 10% Tween-80 aqueous solution (*v*/*v*) to prepare concentrated stock solutions that allow low gavage volumes to feed animals. A maximum volume of 500 μL was established for the gavage treatment in order to avoid regurgitation or stomach discomfort of the animals. Depending on the animal weights (determined weekly), proper volumes of stock solutions were administered to the animals, to reach, based on manufacturer information and our ASTA determinations, the following contents:

(i) CONTROL: 0 mg EPA/kg BW + 0 mg DHA/kg BW + 0 mg ASTA/kg BW;
(ii) FO: 10 mg EPA/kg BW + 7 mg DHA/kg BW + 0 mg ASTA/kg BW;
(iii) ASTA: 0 mg EPA/kg BW + 0 mg DHA/kg BW + 1 mg ASTA/kg BW;
(iv) FO + ASTA: 10 mg EPA/kg BW + 7 mg DHA/kg BW + 1 mg ASTA/kg BW;
(v) KO: 10 mg EPA/kg BW + 4.7 mg DHA/kg BW + 0.0072 mg ASTA/kg BW.

Although additional antioxidants as ascorbate, tocopherols and other carotenoids are present in natural products, their contribution in the total antioxidant capacity of gavage solutions is minor if compared to the prevalent ASTA or (*n*-3)/PUFA components. In addition, we also discarded a significant contribution of (*n*-3)/PUFAs (or antioxidants) from rat chow.

### 4.4. Cerebellar Homogenates

Rat brains were quickly removed after decapitation and washed briefly in 0.1 M phosphate buffer, pH 7.4. Cerebellum was carefully excised and ground in a Potter apparatus with 2.5 mL of 0.1 M phosphate buffer, pH 7.4, under ice-water bath for 3 min. Debris was removed from homogenates by centrifugation at 2.5× *g* for 10 min (4 °C) and fresh (clear) homogenates were kept in ice-water bath for immediate enzyme determinations, or stocked in freezer −80 °C for further biochemical analyses.

#### 4.4.1. Iron Content

Total iron in brain tissues was estimated by the formation of a Fe^2+^: bipyridyl complex after incubation for 2 h at 37 °C in the presence of ascorbic acid (20 mg/mL) and pepsin (10 mg/mL) [60].

#### 4.4.2. Trolox-Equivalent and Ferric-Reducing Antioxidant Capacities

The antioxidant activity in cerebellar homogenates was measured by Trolox-equivalent antioxidant capacity assay (TEAC) and ferric-reducing activity (FRAP). The TEAC assay quantifies the decomposition rate of the aqueous-formed radical 2,2′-azinobis(3-ethylbenzothiazoline-6-sulfonate), ABTS^•−^, by spectrophotometry at 734 nm, in the presence of biological samples using Trolox, a water-soluble derivative of α-tocopherol, as standard [61]. The FRAP assay measures rates of ferric ion reduction (by chelation of formed ferrous ions with 2,3-bis(2-pyridyl)-pyrazine) catalyzed by antioxidants in samples, and detected by absorbance kinetics at 593 nm, for 4 min, in a 96-well microplate reader (SpectraMax M5; Molecular Devices, Sunnyvale, CA, USA) [62,63].

#### 4.4.3. Antioxidant Enzyme Activities

Enzyme activities of superoxide dismutase (total and mitochondrial MnSOD), catalase (CAT), glutathione peroxidase (GPX), and glutathione reductase (GR) activities were determined in cerebellar samples by spectrophotometry techniques adapted for the microplate reader SpectraMax M5, Molecular Devices (Silicon Valley, CA, USA). Briefly, SOD activity was measured at 540 nm by monitoring the linear first-order reduction of O_2_^•−^ radicals by nitroblue tetrazolium (NBT) for 3 min [64]. The activity of the mitochondrial isoform MnSOD was determined by inhibiting cytosolic/extracellular CuZnSOD with 3 mM KCN in the reaction system. Absorbance decay at 240 nm corresponded to H_2_O_2_ consumption by CAT activity present in biological samples [65]. The oxidation of β-NADPH was monitored by absorbance at 340 nm (ε = 6.2 × 10^3^ M^−1^·cm^−1^) in order to measure enzyme activities of GPX and GR [66,67]. Protein determinations followed the analytical protocol described by Bradford (1976), using bovine serum albumin (BSA) as a standard [68].

#### 4.4.4. Indexes of Lipid and Protein Oxidation

Protein fractions were isolated from homogenates by precipitation in 10% trichloroacetic acid in an ice-water bath. After washing once with 0.30 M HClO_4_, 5 mM EDTA and 0.06% 2,2′-bipyridine (*w*/*v*) solution, and twice with an organic mixture (1:1 ethyl acetate: ethanol; *v*/*v*), the protein pellet was dried in vacuum and then subsequently dissolved in 6.0 M guanidine.HCl. Protein sulfhydryl groups(–SH) were detected at 412 nm by the formation of yellowish derivatives with 4 mM 5,5′-dithio-bis(2-nitrobenzoic acid) and quantified using purified glutathione (GSH) as a standard [69]. Unspecific adduct formation was discarded by preliminary sulfhydryl-blockage with 10 mM *N*-ethylmaleimide solution (blank). The extension of lipid peroxidation in cerebellum was evaluated by the thiobarbituric acid reactive substances assay (TBARS). Progressive lipid oxidation in crude homogenates was blocked by adding 20 μL of 4% butylated hydroxytoluene-ethanolic solution. Pinkish TBARS adducts were measured at 535 nm after reaction with 0.25% thiobarbituric acid in 0.25 M HCl and 1% Triton X-100, at 100 °C, for 15 min (blanks lack thiobarbituric acid). Malondialdehyde prepared by acid hydrolysis of 1,1′,2,2′-tetraethoxypropane (TEP) was used as a standard [70].

### 4.5. Motor Cortex and Corpus Callosum

#### 4.5.1. Cytokines Determination

Cytokine concentrations were measured in the motor cortex (molecular and granular layers) of KO-fed Wistar rats in order to monitor the baseline inflammatory scenario upon supplementation (*n* = 3 for both control and KO-fed animals). Levels of tumor necrosis factor-alpha (TNFα), interleukin-6 and 1-beta (IL-6 and IL-1β, respectively), vascular endothelial growth factor (VEGF, stimulus for monocyte/macrophage migration), macrophage inflammation protein (MIP1α), cytokine-induced neutrophil chemoattractant-1 (CINC-1, a chemokine that activates neutrophil infiltration), and l-selectin (l-SEL, an adhesion/homing receptor involved in lymphocyte-endothelial cell interactions) were determined by ELISA, according to the manufacturer’s instructions (DuoSet Kit: Quantikine, R&D System, Minneapolis, MN, USA). The linearity of ELISA methods of all parameters was within the (25–600 pg/mL) range, except for TNF-α (6–1000 pg/mL) and IL-1β (5–250 pg/mL), which include the range of sample determinations. All correlation coefficients of standard curves were in the range of 0.95 to 0.99, whereas intra-assay coefficients of variance were 3%–5%, and inter-assay coefficients of variance were 8%–10%.

#### 4.5.2. Immunohistochemistry

The expression of the astrocyte marker, glial fibrillary acidic protein (GFAP), was analyzed using immunohistochemical staining in the motor cortex (molecular and granular layers) of control and KO-fed rats (*n* =3, each group). The rats were anaesthetized and submitted to intracardiac perfusion with buffered 10% formaldehyde solution. Their brains were then removed and kept for 3 days in the same fixing solution. Coronal sections from the motor cortex were mounted on silanized slides and submitted to GFAP immunostaining using the avidin-biotin peroxidase complex (ABC) method. Briefly, the sections were deparaffinized in xylene and rehydrated in a crescent graded series of ethanol solutions. Antigen retrieval was done by transferring the slides to 10 mM sodium citrate buffer (pH 6.0) at 95 °C for 20 min. Endogenous peroxidase was blocked by 3% hydrogen peroxide for 10 min at room temperature. Two washes with Tris/HCl buffer pH 6.0 (Wash buffer 10×, S3006, Dako, Glostrup, Danmark) were done between incubations. Polyclonal rabbit anti-GFAP immunoglobulin (Z0334, Dako, Glostrup, Danmark), at a dilution of 1:1000, was used as primary antibody, for 16 hour at 4 °C, followed by the application of biotinylated secondary antibody (Dako Universal LSABTM 2 System—HRP, K0690, Glostrup, Danmark), according to the manufacturer’s instructions. Immunoreactivity was visualized by incubating the sections in a solution containing 0.1% diaminobenzidine (DAB, K3467, Dako, Glostrup, Danmark). Sections were then counterstained by Harris’ modified hematoxylin solution, dehydrated and mounted in Entellan (Merck, Darmstadt, Germany). Astrocytic evaluation was done in the molecular and granular layers of the motor cortex and in the corpus calllosum of animals from both groups using a computerized image analysis system (Image-Pro-Plus 4.5, Media Cybernetics, Silver Spring, MD, USA), measuring by colorimetry the area stained brown in a total area of 302,952.5 µm^2^. Negative controls for immunostaining (sections lacking primary antibody application) were done. Data were analyzed by *t* test and statistical significance was set at *p* < 0.05.

### 4.6. Statistical Analysis

All data are presented as the mean values of, at least, triplicates with their standard deviation (MEAN ± SD). Except for assays in immunohistochemistry assays (*n* = 3), all biochemical assays were conducted with 6 or more animals per group (*n* ≥ 6), and data were analyzed as two independent means by the one-tailed *t*-Student’s test followed by Tukey’s post-test (available at Social Science Statistics home page, www.socscistatistics.com).

## 5. Conclusions

Minor redox variations were observed in the cerebellum of Wistar rats supplemented for 45 days with fish oil (FO), ASTA-rich algal biomass (A), fish oil + algal biomass (FO + A), or krill oil (KO). Significant changes in redox metabolism were only observed upon ASTA supplementation, which reinforces the antioxidant properties of this marine carotenoid and putatively suggests its mitochondrial-centered action in mammal neurons. Compared to previous studies from our group, the cerebellum, brain segment strongly associated with neuro-motor control, is less prone to harmful oxidation processes than other cathecolaminergic-rich regions, although we still do not know to what extent. Krill oil, supposedly an alternative source of essential (*n*-3)/PUFAs and ASTA, imposed mild astrocyte activation in motor cortex and corpus callosum of Wistar rats, although no redox or inflammatory index was significantly altered with long-term KO oil supplementation.

Up to now, there is no experimental evidence that FO, ASTA-rich algal biomass, the combination of them, or krill oil supplementation drastically alter the baseline redox or neuro-inflammatory conditions within neuromotor-associated brain regions in animal models.

## Supplementary Files

Supplementary File 1
