# Supplementary material for: Redox Status and Neuro Inflammation Indexes in Cerebellum and Motor Cortex of Wistar Rats Supplemented with Natural Sources of Omega-3 Fatty Acids and Astaxanthin: Fish Oil, Krill Oil, and Algal Biomass
Source: Mar Drugs. 2015 Sep 28;13(10):6117–37. doi: 10.3390/md13106117 (PMC4626682; doi:10.3390/md13106117)
Supplement: Supplementary File 1 [file marinedrugs-13-06117-s001.docx]

Supplementary Materials

Additional Information of Supplement Composition

Fish oil capsules were purchased as Corabion HC 550 supplements (Merck, Relthy Laboratórios Ltda, Indaiatuba, Brazil). Each fish oil (FO) capsule of 550 mg contains 4 kcal (19 kJ), 0.8 mg of mixed tocopherols, and 0.5 g of total fat, which 0.1 g are from saturated fats, 0.1 g from monounsaturated fats (mostly palmitoleic and oleic acids), and 0.3 g of polyunsaturated fatty acids (181 mg EPA and
120 mg DHA).

AstaREAL A1010 biomass was a donation from the Swedish company BioReal AB (Gustavsberg, Sweden), a subsidiary of BioReal Inc. (Hawaii, HI, USA) and part of the pharmaceutical Group Fuji Chemical Industry Co. (Toyama, Japan). AstaREAL A1010 is an astaxanthin-rich natural *Haematococcus pluvialis* product containing 42% of crude fat, 10% of crude protein, 40% of carbohydrates, and 4% of ashes (information provided by BioReal AB). Analytic determination of the carotenoid content shows that AstaREAL A1010 contains 5.0%–5.6% of pure ASTA (3.9% as monoesters, 0.9% as diesters, and 0.1% in free form), 0.02 % lutein/zeaxanthin, 0.02% adonirubin, 0.02% cantaxanthin, 0.02% β-carotene, and 0.1% others. Although ascorbic acid and mixed tocopherols are also residually present in AstaREAL A1010, their contribution is minor in the total antioxidant capacity of gavage solutions if compared to the prevalent ASTA component.

Krill oil was purchased from Mega Red^®^ Extra Strength 500 (Schiff Vitamins, Parsippany, NJ, USA). According to manufacturers, 1 softgel of Mega Red Extra Strength contains 500 mg of purified krill oil (0.5 g total fat, 5 kcal), 115 mg of total omega-3 fatty acids (64 mg EPA, and 30 mg DHA), and 167 mg total phospholipids. No information was provided by manufacturers about the ASTA content in Mega Red softgels. Based on the literature, frozen krill specimens contain 3–4 mg of total carotenoids/100 g frozen krill (>80% of total carotenoids is ASTA) [1]. Uni- or multi-step organic solvent procedures yield around 7–14 g krill oil/100 g krill biomass [2]. Therefore, considering 80% of ASTA in total carotenoid content of krill oil (lower theoretical content) it is expected to find 0.17–0.46 mg ASTA/g krill oil. Repeating the same procedures used here to quantify ASTA in AstaREAL biomass, we actually observed a relatively lower ASTA content in krill oil: 0.092 mg ASTA/g krill oil.

References

1. Yamaguchi, K.; Miki, W.; Toriu, W.; Kondo, Y.; Murakami, M.; Konosu, S.; Satake, M.;
   Fujta, T. The composition of carotenoid pigments in the Antarctic krill *Euphausia superba*.
   *Nippon Suisan Gakkaishi* **1983**, *49*, 1411–1415.
2. Gigliotti, J.C.; Davenport, M.P.; Beamer, S.K.; Tou, J.C.; Jaczynski, J. Extraction and characterisation of lipids from Antarctic krill (*Euphausia superba*). *Food Chem.* **2011**, *125*, 1028–1036.

© 2015 by the authors; licensee MDPI, Basel, Switzerland. This article is an open access article distributed under the terms and conditions of the Creative Commons Attribution license (http://creativecommons.org/licenses/by/4.0/).
